# Examining public knowledge and preferences for adult preventive services coverage

**DOI:** 10.1371/journal.pone.0189661

**Published:** 2017-12-20

**Authors:** Jessica A. R. Williams, Selena E. Ortiz

**Affiliations:** 1 Department of Health Management and Policy, University of Kansas School of Medicine, Kansas City, Kansas, United States of America; 2 Department of Health Policy and Administration, College of Health and Human Development, The Pennsylvania State University, University Park, Pennsylvania, United States of America; University of Tennessee Health Science Center, UNITED STATES

## Abstract

**Introduction:**

To examine (1) what individuals know about the existing adult preventive service coverage provisions of the Affordable Care Act (ACA), and (2) which preventive services individuals think should be covered without cost sharing.

**Methods:**

An online panel from Survey Monkey was used to obtain a sample of 2,990 adults age 18 and older in March 2015, analyzed 2015–2017. A 17-item survey instrument was designed and used to evaluate respondents’ knowledge of the adult preventive services provision of the ACA. Additionally, we asked whether various preventive services should be covered. The data include age, sex, race/ethnicity, and educational attainment as well as measures of political ideology, previous insurance status, the number of chronic conditions, and usual source of care.

**Results:**

Respondents correctly answered 38.6% of the questions about existing coverage under the ACA, while on average respondents thought 12.1 of 15 preventive services should be covered (SD 3.5). Respondents were more knowledgeable about coverage for routine screenings, such as blood pressure (63.4% correct) than potentially stigmatizing screenings, such as for alcohol misuse (28.8% correct). Blood pressure screening received the highest support of coverage (89.8%) while coverage of gym memberships received the lowest support (59.4%). Individuals with conservative ideologies thought fewer services on average should be covered, but the difference was small—around one service less than those with liberal ideologies.

**Conclusions:**

Overwhelmingly, individuals think that most preventive services should be covered without cost sharing. Despite several years of coverage for preventive services, there is still confusion and lack of knowledge about which services are covered.

## Introduction

Seven years after it was signed into law, the Patient Protection and Affordable Care Act (PPACA, hereafter ACA) has been the subject of several repeal attempts and an executive order to halt its enforcement and implementation as much as legally allowed.[1] As of now, there remains considerable speculation about the political or legal steps that will be taken to weaken the ACA; the particular aspects of the ACA that will be revised or repealed, and the health care proposals, if any, that will replace it.[2, 3] This uncertainty and the potential changes or repeals will likely disrupt healthcare for millions of Americans. [3–5]

The ACA’s primary goal was to expand health insurance coverage, while its secondary goals were to control the growth of health care costs and improve the delivery system. While significant problems with the ACA persist, including perverse incentives in the insurance and labor markets[6], it has been far-reaching in its intended effects of expanding health insurance coverage and increasing health benefits, rights, and protections to consumers.[7, 8] The right to appeal health insurance decisions, inability to be denied coverage for pre-existing conditions, and access to preventive health care at no-out-of-pocket cost, exemplify the types of benefits guaranteed not only for individuals and families receiving their care as a result of state Medicaid expansions or through a plan purchased via an ACA insurance exchange, but also for those who receive their health care through an employer-sponsored group health plan or private insurance company.[9]

The ACA provisions related to the use of preventive services have been the target of both media coverage and academic publications.[10–13] But what, exactly, do people know about the ACA’s coverage provisions for preventive services? Which services do people think should be covered? These questions are of particular importance given the precarious state of the ACA and the potential to shape future replacement policies. Recent evidence suggests that only 19.6% of people have heard of “clinical preventive services” as a kind of healthcare.[14] The ACA requires new employer-sponsored group health plans and private health insurance policies to provide coverage without cost sharing for certain preventive services based on recommendations by some national-level institutions, such as the U.S. Preventive Services Task Force (USPSTF) and the Advisory Committee on Immunization Practices.[11] These preventive services were selected because they have “high certainty that the net benefit is moderate or there is moderate certainty that the net benefit is moderate to substantial.”[15, 16] Conceptual models, such as the Grossman model of health production and the Andersen model of health services utilization, suggest that reducing the price of preventive services so that the monetary cost is zero should increase utilization, even though transportation and opportunity costs still exist.[17, 18] Several studies have shown that uptake of many—but not all—preventive services increases when cost sharing is reduced.[19–21]

Confusion about the specifics of health insurance coverage is not unique to the ACA, or to any other new changes to health insurance. Indeed, studies have shown that although consumers have some knowledge of what their health insurance plans cover, knowledge about specific details is often poor.[22–24] Previously uninsured individuals are less likely to know what services are covered by their plan.[22] According to Lantz and colleagues, only 36.4% of adults reported knowing that the ACA requires coverage of certain preventive services without cost sharing.[14] This study assesses knowledge about the *types* of adult preventive services covered without cost sharing. We focus on the clinical preventive services rated A and B by the USPSTF because they are broadly applicable to adults of varying ages and health statuses. Also, we examine the associations between the number and category of adult preventive services people think *should be covered without cost sharing*, political ideology and source of health insurance coverage. We are interested in these particular associations given the polarized, political views of the ACA in general, and because there is a clear sense that people’s views about health coverage are strongly associated with their political ideology and personal experiences.[25–28] For example, would respondents receiving their health insurance through their employer prefer more or less preventive services covered without cost sharing compared to respondents receiving their health insurance through Medicaid or on the marketplace? Results from these analyses could shed some insight on the preferences of recipients of employer-based health insurance (EBHI), a population that is sometimes inaccurately considered exempt from the uncertainty of future proposals that repeal and replace the ACA. The interests of these individuals may be of particular interest to policymakers throughout their deliberations on proposals to replace the ACA, especially regarding those that may decrease the number and type of health services employers are obliged to cover for their employees and whether or not mandates are kept.

## Methods

### Study sample

The study data are from an online, cross-sectional, population-based survey we conducted in March 2015 (N = 2990) and analyzed between 2015 through 2017. SurveyMonkey administered the survey through its Audience Platform and SurveyMonkey Contribute (SMC) online research panel, a service provided by SurveyMonkey to assist customers in reaching a targeted audience for their surveys. The SMC online research panel was recruited from over 30 million people who answer SurveyMonkey surveys each month. SurveyMonkey automatically computes the number of panelists to invite to take the survey experiment based on 1) the number of finished responses requested; 2) the response rates of individual survey respondents; and 3) the availability of survey respondents who meet the study’s targeting criteria (aged 18 or older). This study was approved by the Harvard School of Public Health IRB (#IRB14-4131).

### Measures

A 17-item survey instrument was designed in part to evaluate respondents’ knowledge of the preventive services coverage provision of the ACA (S1 Appendix). Respondents were explicitly asked to identify, to the best of their knowledge, whether particular services were covered without cost sharing for adults under the ACA (*Covered/Not Covered/Unsure or Don’t Know*). The covered services discussed in this study are all listed prominently on healthcare.gov. To guard against the possibility of selecting all covered or all uncovered for the knowledge questions, respondents were also asked about services that are preventive but not covered under the ACA (e.g. telephone nurse help lines) and services that are sometimes covered but have cost sharing in many existing health insurance plans (e.g. prescription drugs).[29] The following services were included in the knowledge index: colorectal cancer screening, blood pressure screening, type 2 diabetes screening, high cholesterol screening, depression screening, healthy diet counseling, influenza shots, subsidized gym membership, nurse advice telephone line, stress management counseling, sexually transmitted infection counseling, obesity screening, alcohol misuse screening, tobacco use screening, eye exam, hearing test, and allergy shots. Response randomization for the order of all responses was used to alleviate order and survey-fatigue bias.

Health insurance status, main source of health insurance, usual source of care, sociodemographic questions and number of self-reported chronic conditions were measured using validated questions from the California Health Interview Survey.[30] The question about political ideology comes from the American National Elections Studies survey. [31]

### Statistical analysis

The knowledge index was created by tallying the number of correct answers, or, the number of times respondents correctly identified services that were covered and services that were not covered. Answers of *"unsure or don’t know"* were counted as incorrect. The concept of cost sharing was discussed within the survey per the following statement: *“The Affordable Care Act requires health insurance plans to provide certain preventive services to adults without a copayment or coinsurance*.*”* A total of 17 questions were used to measure respondents’ knowledge of preventive services coverage, yielding a theoretical range of [0, 17]. The number of services respondents thought *should be* covered were summed from the items in the knowledge index (15 possible services). When summing the number of services respondents thought *should be* covered, answers of “*unsure or don’t know*” were assigned a value of 0.5.

To investigate whether political ideology or source of health insurance was associated with the number of preventive health services by category people think should be covered without cost sharing, two separate indices were created. The first index (aka ‘typical’) ranged from 0 to 5 and measured adult preventive services including colorectal cancer screening, blood pressure screening, diabetes (type 2) screening, cholesterol screening, and flu shots. The second index (aka ‘personal health behavior’) ranged from 0 to 5 and measured the number of adult preventive services that are stereotypically categorized as those associated with poor health behaviors, including screenings for obesity, alcohol abuse, tobacco use, sexually transmitted infections and healthy diet counseling.[32]

Two primary predictors were investigated: political ideology, which was measured on a 7-point scale (*extremely conservative*, *conservative*, *slightly conservative*, *moderate/middle of the road*, *slightly liberal*, *liberal*, and *extremely liberal*), and main source of health insurance coverage (*employer-based (own)*, *employer-based (spouse)*, *direct purchase via private insurance company or a state or federal marketplace*, *Medicare*, *Medicaid*, or *other*). The models also adjusted for potential sociodemographic and health confounders. Sociodemographic characteristics included age, gender, race/ethnicity (*American Indian/Alaskan Native*, *Asian/Pacific Islander*, *Black/African American*, *Latino*, and *White/Caucasian*), educational attainment (l*ess high school diploma*, *high school diploma*, *some college*, *bachelor’s degree*, or *graduate degree*), marital status (*married*, *divorced*, *separated*, *widowed*, or *never married*) and employment status (*employed (1–40 hours)*, *employed (>40 hours)*, *not employed/looking for work*, *not employed/not looking for work*, *retired*, or *disabled*). Health characteristics included self-reported health status (*excellent*, *very good*, *good*, *fair*, or *poor*), health insurance coverage status in the last 12 months (*yes/no/unsure or don’t know*), and whether have usual source of health care (*yes/no/unsure or don’t know*).

Univariate statistics were calculated to describe the sample and examine individual’s knowledge of preventive services without cost sharing. Multivariable analyses using ordinary least squares (OLS) were conducted to examine associations using Stata 14.0.[33]

## Results

Descriptive statistics for the sample and the U.S. population are given in Table 1. Overall, the SMC sample was slightly younger, had higher education levels, and had fewer Hispanic Americans compared to the U.S. population. These characteristics were controlled for in all of the analyses.

**Table 1:** Sample characteristics and comparison to U.S. non-institutionalized civilian population 18 years or older.

	Sample	U.S. Population^a^
**Political Ideology (%)**		
Extremely Conservative	8.2	4
Conservative	16.3	18
Slightly Conservative	11.5	14
Moderate, middle of the road	39.3	32
Slightly Liberal	8.9	11
Liberal	10.9	10
Extremely Liberal	4.9	3
**Have Health Insurance (%)**		
Yes	86.3	89.3
No	12.3	10.8
Unsure/Don’t know	1.4	
**Have a Usual Source of Care (%)**	79.1	85.3
**Education**		
Less than High School	3.6	13.0
High School Graduate	24.9	27.6
Some College	31.1	28.9
College Graduate	27.2	19.0
Some or Completed Graduate School	13.2	11.6
**Work (%)**		
Employed	64.0	59.3
Not Employed	21.6	18.8
Retired	5.7	15.4
Disabled, not able to work	8.8	6.5
**Marital Status (%)**		
Married	47.6	47.5
Divorced, Separated, Widowed	14.9	19.1
Never Married	37.6	33.6
**Age (%)**		
18–29	25.6	20.7
30–44	34.5	19.3
45–59	32.7	20.3
60+	7.2	20.8
**Female (%)**	52.3	51.0
**Race/Ethnicity (%)**		
American Indian or Alaskan Native	1.44	0.7
Asian / Pacific Islander	5.2	5.6
Black or African American	11.3	12.4
Hispanic American	7.4	17.6
White / Caucasian	74.7	61.6
**Health Status (%)**		
Excellent & Very Good	51.8	60.5
Good	14.0	26.9
Fair & Poor	34.3	12.6

Health insurance, usual source of care, and health status data come from the 2015 National Health Interview Survey; Education, marital status, age, gender, and race/ethnicity data come from the 2015 American Community Survey; Employment data come from the Bureau of Labor Statistics (2015 with retirement estimates from 2014).

^a^. Political ideology data come from the American National Election Studies 2012 Survey.

### Knowledge of current coverage for preventive services

**Fig 1:**
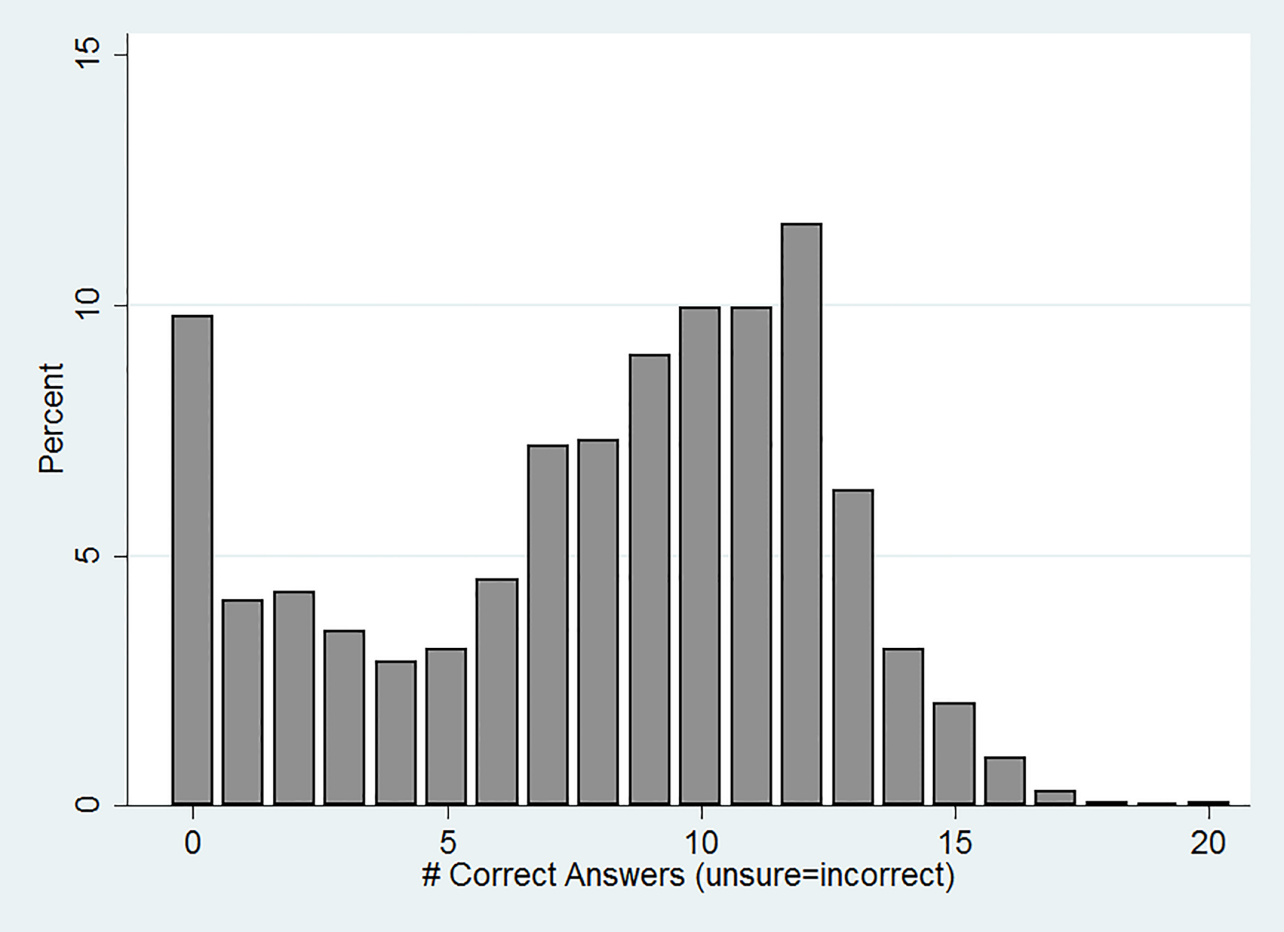
Distribution of Scores on ACA Preventive Service Knowledge Index. This figure shows the distribution of scores on the ACA Preventive Service Knowledge Index (N = 2990).

The knowledge index had an empirical range of [0, 16] with a mean of 6.6 (SD 3.9), see Table 2. As Fig 1 shows, the distribution of total scores appears to have two distinct peaks: 10 (*n* = 482) and 0 (*n* = 480). The majority, 60.3%, of individuals answered *“don’t know or unsure”* to at least one question (on average 5.7 questions per respondent, SD 6.5, Table 2). On average, respondents were least likely to correctly answer questions about whether hearing screening (21.6%) and nurse advice telephone line (22.7%) were covered without cost sharing, neither of which are (Table 3). A majority of respondents were more likely to correctly identify blood pressure screening (63.7%), flu shots (62.1%), and cholesterol screening (56.8%) as being covered without cost sharing.

**Table 2:** Means and standard deviations of key constructs.

Construct	Mean (Standard Deviation)
Knowledge Index [0, 16]	6.6 (3.9)
Number of questions answered with “don’t know or unsure” per respondent	5.7 (6.5)
Number of the 17 adult preventive services examined that *should* be covered without cost sharing per respondent	13.3 (4.0)
Number of ‘typical’ adult preventive services examined that *should* be covered without cost sharing per respondent^a^	4.88 (2.1)
Number of ‘personal health behavior’ adult preventive services examined that *should* be covered without cost sharing per respondent^b^	4.39 (1.18)

^a^. Cancer screening, blood pressure screening, diabetes (type 2) screening, cholesterol screening, and flu shots.

^b^. Screenings for obesity, alcohol abuse, tobacco use, and sexually transmitted infections and healthy diet counseling.

**Table 3:** Comparing the percent of people who think services are covered by the ACA with the percent of people who think services should be covered.

	% of people who think this service *is* a covered benefit under the ACA/Obamacare	% of people who think this service *should* be a covered benefit	Difference
Colorectal cancer screening covered?	48.6	91.3	42.7
Blood pressure screening covered?	63.7	93.7	30.0
Diabetes (type 2) screening covered?	53.4	92.4	39.0
Cholesterol screening covered?	56.8	91.4	34.6
Depression screening covered?	37.8	87.5	49.7
Influenza shot covered?	62.1	90.6	28.5
Obesity screening covered?	35.2	78.6	43.4
Alcohol misuse screening covered?	28.0	69.6	41.6
Sexually transmitted infection (STI) prevention counseling covered?	39.3	80.7	41.4
Healthy diet counseling covered? ^a^	69.9	78.6	8.7
Subsidized gym membership covered? ^a^	56.6	61.3	4.7
Nurse advice telephone line covered? ^a^	77.3	83.2	5.9
Stress management counseling covered? ^a^	68.8	79.7	10.9
Eye exams are covered? ^a^	71.6	90.6	19.0
Hearing screening covered? ^a^	78.4	90.1	11.7
Allergy shots covered? ^a^	74.1	84.9	10.8
Tobacco use screening covered?	27.9	62.8	34.9

^a^. Services not covered at the time of the survey.

### How many preventive services should be covered?

For each preventive service, Table 3 lists: (1) the percentage of respondents who thought it was covered without cost sharing under the ACA and (2) the percentage of respondents who thought it *should* be covered without cost sharing. More often, a majority of respondents incorrectly identified non-covered preventive services as being covered with no cost sharing under the ACA, such as nurse advice telephone line (77.3%). Furthermore, a majority of respondents were unlikely to correctly identify covered but health behavior related services, such as alcohol misuse screening (28.0%).

Among the 17 adult preventive services examined, on average, a majority of respondents thought that most services (13.3, SD 4.0) should be covered without cost sharing (see Table 2). The percentage of respondents who thought that an adult preventive service should be covered without cost sharing was very high for routine preventive services, see Table 3, such as screening for high blood pressure (93.7%) and type 2 diabetes (92.4%).

### How many and what types of preventive services should be covered without cost sharing?

In an adjusted multivariable regression model with the number of preventive services as a key outcome (Table 4), there were significant differences by political ideology. Compared to moderate/middle of the road respondents, conservative and extremely conservative respondents significantly preferred less than 1 fewer services be covered without cost sharing, adjusting for all other factors. Conversely, liberal and extremely liberal respondents preferred an additional 0.52 and 1.09 services be covered, respectively. There were no significant differences in the number of preventive services that people thought should be covered without cost sharing by main source of health insurance, with the exception that people without health insurance (n = 368) or those who reported that they “didn’t know whether they had health insurance” (n = 43) preferred that 1.43 fewer services should be covered than people with their own EBHI.

**Table 4:** Coefficients and 95% CIs for the Linear regression of the number of services that should be covered, typical services, and personal health behavior services. (N = 2989^a^).

	# of Services that *should* be covered: all services	# of Services that *should* be covered: ‘Typical’ Services^b^	# of Services that *should* be covered: ‘Personal Health Behavior Services’^c^
	Coefficient [95% CI]	Coefficient [95% CI]	Coefficient [95% CI]
**Political Ideology (Moderate, middle of the road is reference)**			
Extremely Conservative	-0.79**	-0.29**	-0.24
	**[-1.34,-0.23]**	**[-0.46, -0.13]**	[-0.54, 0.06]
Conservative	-0.95**	-0.17**	-0.55**
	**[-1.36, -0.53]**	**[-0.30, -0.06]**	**[-0.77, -0.32]**
Slightly Conservative	-0.45	-0.11	**-0.25**
	[-0.92, 0.02]	[-0.25, 0.03]	[-0.51, 0.001]
Slightly Liberal	0.2	0.16*	-0.04
	[-0.32, 0.72]	**[0.01, 0.32]**	[-0.32, 0.24]
Liberal	0.52*	0.18*	0.21
	**[0.04, 1.00]**	**[0.04, 0.32]**	[-0.05, 0.47]
Extremely Liberal	1.09**	0.15	0.70**
	**[0.42, 1.76]**	[-0.04, 0.35]	**[0.33, 1.06]**
**Have Health Insurance (Employer Plan (own) is reference)**			
Employer Plan (spouse)	-0.35	-0.08	-0.22
	[-0.85, 0.14]	[-0.22, 0.07]	[-0.49, 0.04]
Medicare	-0.21	-0.09	-0.11
	[-0.80, 0.39]	[-0.27, 0.08]	[-0.43, 0.21]
Medicaid	0.3	0.04	0.11
	[-0.23, 0.82]	[-0.12, 0.19]	[-0.17, 0.40]
Other	0.6	0.12	0.25
	[-0.05, 1.25]	[-0.08, 0.31]	[-0.17, 0.40]
Marketplace plan	-0.19	-0.09	-0.06
	[-0.66, 0.28]	[-0.23, 0.05]	[-0.31, 0.20]
No insurance/don’t know	-1.43**	-0.48**	-0.57**
	**[-1.92, -0.93]**	**[-0.63, -0.34]**	**[-0.84, -0.30]**
**Have a Usual Source of Care**	1.06**	0.35**	0.41**
	**[0.70, 1.42]**	**[0.25, 0.46]**	**[0.22, 0.61]**
**Education (Some College is reference)**			
Less than High School	-0.91*	-0.36**	**-0.26**
	**[-1.70, -0.12]**	**[-0.60, -0.14]**	**[-0.69, 0.16]**
High School Graduate	-0.02	0.02	-0.04
	[-0.40, 0.36]	[-0.09, 0.13]	[-0.24, 0.17]
College Graduate	-0.09	0.02	-0.09
	[-0.47, 0.28]	0.02	[-0.29, 0.12]
Some or Completed Graduate School	-0.33	-0.12	-0.06
	[-0.80, 0.14]	[-0.26, 0.02]	[-0.31, 0.20]
**Work (Employed is reference)**			
Not Employed	0.05	0.03	0.002
	[-0.34, 0.44]	[-0.09, 0.14]	[-0.21, 0.21]
Retired	-0.71*	-0.16	-0.36
	**[-1.40, -0.01]**	[-0.36, 0.05]	[-0.74, 0.01]
Disabled, not able to work	0.59*	0.09	0.35*
	[-0.01, 1.19]	[-0.09, 0.26]	[0.02, 0.67]
**Marital Status (Married is reference)**			
Divorced, Separated, Widowed	0.02	-0.06	0.12
	[-0.42, 0.47]	[-0.19, 0.07]	[-0.12, 0.36]
Never Married	-0.28	-0.06	-0.09
	[-0.64, 0.08]	[-0.17, 0.04]	[-0.29, 0.10]
**Age (30–44 is reference)**			
18–29	-0.6*	-0.21**	-0.26*
	**[-1.00, -0.20]**	**[-0.33, -0.10]**	**[-0.48, -0.05]**
45–59	-0.28	0.03	-0.26**
	[-0.63, 0.07]	[-0.07, 0.13]	[-0.45, -0.07]
60+	0.09	0.22*	-0.17
	[-0.54, 0.72]	**[0.03, 0.40]**	[-0.51, 0.17]
**Male**	-0.92**	-0.26**	-0.38**
	**[-1.21, -0.63]**	**[-0.34, -0.17]**	**[-0.54, -0.22]**
**Race/Ethnicity (White/Caucasian is reference)**			
American Indian or Alaskan Native	1.31*	0.30	0.72*
	**[0.13, 2.49]**	[-0.04, 0.65]	**[0.09, 1.36]**
Asian /Pacific Islander	0.29	0.00	0.13
	[-0.36, 0.93]	[-0.19, 0.18]	[-0.22, 0.47]
Black or African American	0.13	-0.04	0.15
	[-0.32, 0.58]	[-0.17, 0.09]	[-0.10, 0.39]
Hispanic American	0.17	0.06	0.09
	[-0.38, 0.71]	[-0.10, 0.22]	[-0.21, 0.38]
**Health Status (Good is reference)**			
Excellent	-0.35	-0.15*	-0.08
	[-0.78, 0.09]	**[-0.28, -0.02]**	[-0.32, 0.16]
Very Good	-0.33*	-0.08	-0.15
	**[-0.67, 0.01]**	[-0.18, 0.02]	[-0.33, 0.04]
Fair	-0.2	0.01	-0.17
	[-0.69, 0.29]	[-0.13, 0.15]	[-0.44, 0.10]
Poor	-0.07	-0.02	-0.03
	[-0.99, 0.85]	[-0.29, 0.24]	[-0.53, 0.47]

Boldface indicates statistical significance (*p<0.05, **p<0.01).

^a^. One person was dropped because they were missing an education value.

^b^. Colorectal cancer screening, blood pressure screening, diabetes (type 2) screening, cholesterol screening, and flu shots.

^c^. Screenings for obesity, alcohol abuse, tobacco use, sexually transmitted infections and healthy diet counseling.

Results in Table 4 suggest that political ideology was also significantly associated with the number of preventive services per category (typical and personal health behavior) that should be covered. In regard to those services categorized as *typical* (i.e. cancer screening, blood pressure screening, diabetes (type 2) screening, cholesterol screening, and flu shots), conservative and extremely conservative respondents preferred 0.17 and 0.29 fewer typical preventive services should be covered without cost sharing, adjusting for all other factors. For comparison, on average people thought that 4.88 services should be covered (SD 2.1, Table 2). Slightly liberal and liberal respondents, however, reported that an additional 0.18 and 0.15 typical preventive services should be covered, respectively, adjusting for all other factors. Respondents with no health insurance or those who reported not knowing whether they had health insurance, preferred that 0.48 fewer services should be covered than people with EBHI, adjusting for all other factors. No other significant differences were found by health insurance type.

With regard to those services categorized as *personal health behavior* (i.e., screenings for obesity, alcohol abuse, tobacco use, and sexually transmitted infections and healthy diet counseling), political ideology was also found to be consequential (on average people thought 4.39 services should be covered, SD 1.18, Table 2). Compared to moderate/middle of the road respondents, conservative respondents significantly preferred that 0.55 fewer personal health behavior preventive services should be covered without cost sharing, while extremely liberal respondents significantly preferred 0.70 more personal health behavior preventive services be covered, adjusting for all other factors. And, as with the previous models, people without health insurance or those who reported that they “didn’t know whether they had health insurance” preferred that 0.57 fewer services should be covered than people with EBHI, adjusting for all other factors.

## Discussion

For ‘typical’ screenings, such as blood pressure, respondents were more knowledgeable about coverage than rarer or potentially stigmatizing screenings, such as for alcohol misuse.[34] We consider several reasons why this may be the case. For example, it is possible that stigma that may be ascribed to particular types of screening and personal health behaviors may contribute to public misconception.[35–37] ACA guidelines and the implementation of those guidelines may also contribute to low public knowledge. For example, while health insurers are currently permitted to impose a tobacco surcharge to smokers, they are also required to provide the full realm of FDA-approved tobacco cessation medications without cost sharing. However, a recent study found that only one state ensured access to smoking cessation programs as the ACA required.[38] Furthermore, of the 348 health insurance issuers available through exchanges, only 17% explicitly advertised no cost-sharing cessation programs.[38]

Poor knowledge may also be due to a misperception among the public that coverage is available for screenings about ‘unobserved’ conditions, such as hypertension, but unavailable for conditions that are more visible or perceived as being related to personal health behaviors, such as obesity. We recommend future studies examine public understanding about the preventive services covered without cost sharing. Mixed-method approaches are well suited to exploring these types of questions.

Are people as polarized as they seem to be about health care? When we investigate the association of political ideology with beliefs about preventive services and cost sharing, we find results that are statistically significant but so small as to be politically irrelevant (differences of about one service with a standard deviation of four services). These findings correspond with a recent Kaiser Health Tracking poll that found that Republicans and Democrats share common perspectives on many provisions of the ACA, particularly no-cost sharing of preventive services. [39] Among Republicans, 77% have a favorable view towards the elimination of out-of-pocket costs for preventive services. Although Democratic views are more favorable (90%) than Republican views, this finding underscores the fact that major aspects of the health law are quite popular across party lines. Proposed plans to replace the ACA will need to consider the bi-partisan support of the preventive services coverage provision.

Finally, with the exception of individuals who were uninsured or those who reported not knowing what their main source of health insurance coverage was, health insurance type was not significantly associated with preferences regarding the number or category of adult preventive services that people think should be covered without cost sharing compared to respondents receiving their health insurance through their employer. We are unsure as to why the uninsured preferred fewer preventive services be covered. It could be that preferences for certain types of medical care consumption are lower in this population than those receiving EBHI.[40] Qualitative studies could investigate how preferences for particular types of adult preventive care services differ by health insurance coverage type, while mixed-methods approaches could examine whether preferences among the uninsured vary by demographic characteristics.

### Limitations

It is possible that participants did not distinguish between general coverage for medical, dental, and other health services and preventive services newly mandated to be covered without cost sharing under the ACA. To ameliorate this concern the study instrument included very specific prompts about the ACA, type of insurance, and the services being considered.

The study was unable to control for the health literacy of survey respondents. Level of survey readability could have resulted in misunderstandings of definitions and other text passages, as well as in significant differences in knowledge. Future studies should investigate the relationship between health literacy and knowledge of preventive services coverage.

The sample was not randomly selected, and while it was similar to the U.S. population regarding some demographic characteristics, it contained relatively more highly educated people, more white people, and more people under the age of 44. Given the limited evidence available in the area, the survey still provides useful information because of the SMC panel’s comparability with other national samples and the timeliness of the information provided.

### Conclusion

In general, people want preventive services to be covered without cost sharing. Still, even though many of the services have been covered on non-grandfathered plan years since September 2010, most people do not know they are covered. Research evaluating the effect of the increased coverage on service utilization should consider whether differential knowledge about services might affect individual responses to changes in price. Future attempts at health insurance legislation may do well to include coverage for preventive services, they appear to be popular and have the additional benefit of potentially improving the health of the population as a whole—assuming you can get people to use them! Therefore, whether the ACA remains ‘as is,’ or, if proposals that retain the provision of no cost sharing of preventive services are ultimately adopted, new and effective strategies that can increase public knowledge about the availability of preventive services are essential.

## Supporting information

S1 Appendix Survey questions used to create the knowledge index. These are the survey questions that were used to create the Knowledge Index.
(PDF)

S1 Data Data used for the analysis. (XLSX)
