# Supplementary material for: Examining public knowledge and preferences for adult preventive services coverage
Source: PLoS One. 2017 Dec 20;12(12):e0189661. doi: 10.1371/journal.pone.0189661 (PMC5738055; doi:10.1371/journal.pone.0189661)
Supplement: S1 Appendix — These are the survey questions that were used to create the Knowledge Index. (PDF) [file pone.0189661.s001.pdf]

Appendix Exhibit 1: Survey questions used to create the Knowledge Index

To the best of your knowledge, which of the following adult services do you think are covered without a co-payment or co-insurance? (Please mark all that apply).

	Covered	Not covered	Unsure/Don't know
Colorectal cancer screening	◯	◯	◯
Influenza (flu) shot	◯	◯	◯
Tobacco use screening	◯	◯	◯
Diabetes (Type 2) screening	◯	◯	◯
Cholesterol screening	◯	◯	◯
Alcohol misuse screening	◯	◯	◯
Sexually transmitted infection (STI) prevention counseling	◯	◯	◯
Eye exams	◯	◯	◯
Subsidized gym membership (for overweight or obese individuals only)	◯	◯	◯
Healthy diet counseling	◯	◯	◯
Depression screening	◯	◯	◯
Hearing screening	◯	◯	◯
Stress management counseling	◯	◯	◯
Allergy shots	◯	◯	◯
Blood pressure screening	◯	◯	◯
Nurse advice telephone line	◯	◯	◯
Obesity screening	◯	◯	◯
